# Infectious disease prevalence among unaccompanied minor refugees: Associations with migration journey characteristics

**DOI:** 10.1371/journal.pone.0354114

**Published:** 2026-09-01

**Authors:** Heribert Stich, Angelika Deisling, Fabian Standl

**Affiliations:** 1 Institute for Medical Information Processing, Biometry, and Epidemiology-IBE, University of Munich-LMU, Campus Grosshadern, Munich, Germany; 2 Pettenkofer School of Public Health, Munich, Germany; 3 Department of Public Health Medicine, Landshut, Germany; 4 Institute for Medical Informatics, Biometry and Epidemiology (IMIBE), University Medicine Essen, Essen, Germany; 5 Technical University of Munich, Germany- TUM School of Medicine and Health, Graduate Center of Medicine and Health, Munich, Bavaria, Germany; University College London, UNITED KINGDOM OF GREAT BRITAIN AND NORTHERN IRELAND

## Abstract

**Background:**

We examined infectious disease screening in unaccompanied minors because migration-related exposures may increase the risk of communicable diseases and because early detection enables timely treatment and prevention. However, the relationship between migration journey characteristics and infectious disease prevalence in this population remains poorly understood. We therefore examined infectious disease prevalence among unaccompanied minors upon arrival in Germany and explored its association with migration journey characteristics.

**Methods:**

We conducted a retrospective cross-sectional study using standardized, case-specific health assessments conducted by the Public Health Service in one Bavarian district. Routine data were collected over thirteen consecutive years (2010–2022). We first described the prevalence of detected infections in the full cohort of 237 unaccompanied minors undergoing legally mandated screening, including tuberculosis, hepatitis A, hepatitis B, hepatitis C, scabies, and intestinal parasitic infections. We then analyzed the subgroup of 129 minors with documented migration journey information to describe socio-epidemiological profiles and explore patterns according to number of transit countries, journey duration, and modes of migration.

**Results:**

In the full cohort, at least one infectious disease was detected in 61.2% of minors. Prevalence patterns were broadly similar in the migration-history subgroup, where 65.1% had at least one detected infectious disease. In the subgroup, most unaccompanied minors were male (79.8%) and aged 13.5 to 16.5 years (55.0%). Tuberculosis prevalence was highest among Sub-Saharan African participants (20.0%, 95% CI 11.2–32.7), while hepatitis B predominated in North African and Southeast European minors. Over half (52.9%) passed through three to four countries, and 72.1% used two different modes of travel. Although confidence intervals were wide, infectious disease positivity was numerically higher among minors with longer documented migration journeys.

**Conclusions:**

Infectious disease positivity was common among unaccompanied minors in this cohort and showed similar overall prevalence patterns in the full cohort and the migration-history subgroup. Migration journey characteristics provided useful descriptive context, but observed differences by journey duration were not supported by clear statistical evidence and should be interpreted cautiously given the limited sample size, wide confidence intervals, and potential confounding. These findings support structured health screening after arrival and careful documentation of migration history during clinical assessment.

## Background

Forced migration is a critical public health challenge. Approximately 110 million people worldwide were forcibly displaced in 2023 [1]. A relevant proportion of these were children and adolescents, including unaccompanied minors, that is, individuals younger than 18 years who migrate without parents or legal guardians [2–4]. Unstable living conditions, interrupted healthcare, and migration-related exposures may increase this group’s vulnerability to communicable diseases [5–7]. Although infectious disease prevalence has been documented in broader migrant populations [8–15], the relationship between migration journey characteristics and infection patterns in unaccompanied minors remains poorly characterized. Germany requires standardized health screening for all asylum seekers upon arrival. Depending on age and clinical indication, these examinations include legally required testing for selected infectious diseases such as tuberculosis, hepatitis A, hepatitis B, hepatitis C, and selected parasitic and dermatological infections [16]. We conducted this study over 13 years because relatively few eligible cases presented annually in this region. During the study period, Germany received substantial numbers of asylum seekers, with Bavaria implementing regionally organized reception and health screening structures [17].

## Methods

### Study design

We conducted a retrospective cross-sectional analysis over 13 consecutive years (2010–2022) in the Landshut district of Bavaria, Germany (population ~230,000). We analyzed routinely collected health data supplemented by standardized surveys capturing detailed migration journey and medical history information. The anonymized dataset supporting this study is publicly available via mediatum [18]. The full study population consisted of all 237 unaccompanied minors examined by the Public Health Service between 2010 and 2022. We restricted this analysis to 129 minors with sufficiently documented migration journey information. Individuals without sufficiently documented migration journey information were not included in the migration-specific analyses. Of the full cohort, 108 had insufficiently documented migration history information and were therefore excluded from the migration-specific analysis. The study was approved by the Ethics Committee of the Medical Faculty at Ludwig Maximilian University of Munich (Project No. 23–0150). Verbal informed consent was obtained from all participants and documented by the examining physician. This study is reported in accordance with the STROBE (Strengthening the Reporting of Observational Studies in Epidemiology) guidelines for cross-sectional studies.

### Biomedical examination

In accordance with § 62 of the Asylum Act (AsylG), unaccompanied minors are required by federal law to undergo a health examination by the Public Health Service (PHS) within the first three days of arrival in Germany [19]. Clinicians focus particularly on communicable disease signs, especially dermatological manifestations (itchy redness, nodules, papules) indicating parasitic infections such as scabies. We performed clinical diagnoses and diagnostic procedures according to applicable medical guidelines [16,19–23], established disease definitions [24], and procedural recommendations current at examination [25]. All unaccompanied minors are accompanied to the health department by representatives of the local Youth and Welfare Office. The medical examination team remained consistent throughout the 13-year study period, ensuring continuity and standardization. We documented all findings using standardized forms. We used age-dependent screening procedures. We performed standard serological testing for hepatitis A, hepatitis B, hepatitis C, HIV, and syphilis in children aged 10 years and older in accordance with the regulations and procedures in place during the study period. Tuberculosis screening varied by age: adolescents ≥15 years underwent chest radiography, while younger children received clinical examination and IGRA-based testing per pediatric guidelines. Because screening protocols varied by age, prevalence estimates should be interpreted considering these age-specific differences.

### Biomedical laboratory analyses

Federal regulations require all asylum seekers in Germany to undergo a standard panel of laboratory tests for infectious diseases upon arrival [16]. These include serological testing for hepatitis A (Anti-HAV-IgM for acute, Anti-HAV-IgG for past infection), hepatitis B (HBsAg, Anti-HBc, Anti-HBs, and HBV-DNA-PCR to distinguish between acute and chronic infection), and hepatitis C (Anti-HCV antibodies and HCV-RNA for differentiation of infection status). Additional mandatory screenings included tests for HIV types I and II, syphilis (TPHA test), tuberculosis, and stool examinations to detect bacterial pathogens causing diarrhea, particularly those related to typhoid, paratyphoid, intestinal inflammation, dysentery, and intestinal parasitosis [16].

Hepatitis B screening included serological markers for surface antigen (HBsAg), surface antibodies (anti-HBs), and core antibodies (anti-HBc). The presence of HBsAg indicates active infection, while anti-HBs typically indicates immunity from vaccination or past infection, and anti-HBc indicates previous or ongoing infection. HIV testing is performed using a combined antigen-antibody assay to detect both the HIV p24 antigen and antibodies against HIV I/II. Hepatitis A and C diagnoses were based on the detection of specific antibodies: anti-HAV and anti-HCV, respectively.

For tuberculosis, §36 (4) of the Infection Protection Act (IfSG) mandates that asylum seekers aged 15 years or older receive a chest X-ray upon admission to shared accommodations to rule out infectious pulmonary tuberculosis [16]. Exceptions apply to pregnant women and children under 15 years, for whom a medical certificate must confirm no clinical signs of active TB. In line with the Working Group of the Scientific Medical Societies (AWMF) guidelines for diagnosing tuberculosis in children and adolescents, gamma interferon release assay (IGRA) is used as a suitable alternative screening method [26]. However, IGRA cannot distinguish between active tuberculosis (positive IGRA, abnormal chest X-ray, and bacteriological confirmation in sputum) and latent tuberculosis infection (positive IGRA only) [20,22]. Therefore, for positive IGRA results, a follow-up chest X-ray is performed to exclude active disease. In accordance with the German Asylum Act (Asylgesetz) [27] and clinical guidelines [25], standardized blood tests for hepatitis A, B, C, HIV, and syphilis were conducted for all children aged 10 years and older.

### Supplementary survey on the migration process

We collected migration journey information through structured interviews during the standardized medical assessment. Specific data points included: country of birth, region of origin according to WHO classifications, modes of transportation (air, land, sea), number of transit countries, and total duration of journey in months. For the purposes of this study, these questions were asked more systematically, and responses were documented in greater detail to ensure data quality and consistency. Only individuals who voluntarily provided complete responses to the migration-related questions were included in the final analysis.

### Interview and consent procedures

All interviews were conducted in English whenever possible. If an unaccompanied minor was not sufficiently proficient in English, a certified interpreter fluent in the minor’s native language was brought in prior to the assessment to prevent any communication barriers. This approach aimed to create a safe and supportive environment, particularly for minors who may have experienced psychological trauma or distress. Before the examination, unaccompanied minors were asked about their preferences regarding the assessment setting, for example, whether they wished to have the Youth and Welfare Officer present or whether they preferred to be examined by a male or female physician. As part of the standardized procedure, minors were verbally informed about the purpose (mandatory examination), content, and formalities of the health assessment. Verbal informed consent was obtained in accordance with German asylum law requirements for mandatory health examinations. Minors were accompanied by Youth and Welfare Office representatives throughout.

### Data analysis

Based on the information provided by unaccompanied minors, we calculated the prevalence of key socio-epidemiological characteristics and infectious diseases required by law to be tested, including hepatitis A, B, and C, intestinal parasitosis, scabies, (latent) tuberculosis infection, and pulmonary tuberculosis. Laboratory test results, stool sample findings (coded as 1 = positive, 0 = negative), and the composite variable “infectious disease positive” (defined as testing positive for at least one of the specified infectious diseases: hepatitis A, B, or C, intestinal parasitosis, scabies, or tuberculosis) were recorded as a binary outcomes. The regions of origin were categorized according to WHO regional classifications [21]: South Asia, North Africa, Middle East, Sub-Saharan Africa, and Southeast Europe. We used descriptive statistics to summarize our findings. Categorical variables (such as sex, region of origin, and infection status) are presented as percentages with 95% confidence intervals (95% CI). Continuous variables (such as age and journey duration) are described using means and standard deviations to indicate central tendency and variability. For the main analysis, we calculated point estimates of disease prevalence with corresponding 95% CI using the Wilson score method, which is appropriate for small sample sizes and provides more accurate coverage than traditional normal approximation methods. To allow readers to assess the relationship between the full cohort and the migration-history subgroup, we also reported infection prevalence for the full cohort of 237 minors and compared these estimates descriptively with the corresponding estimates in the subgroup of 129 minors with documented migration journey information. Analyses involving migration journey characteristics were restricted to this subgroup. Migration modes were explored descriptively but are not presented in detail due to small subgroup sizes and heterogeneous patterns. We defined age groups based on the empirical distribution of the study population at the beginning of the project and kept them consistent throughout the observation period to allow comparability over time. Specifically, the youngest group corresponds to the lower quartile (≤25th percentile), the middle group to the interquartile range (25th-75th percentile), and the oldest group to the upper quartile (≥75th percentile).

### Data protection and compliance with ethical guidelines

We conducted this retrospective cross-sectional analysis in full compliance with applicable data protection regulations, under the supervision of the responsible data protection officer, and in accordance with the Declaration of Helsinki (1975), including its most recent revisions. Prior to submission to the Ethics Committee, the study received formal approval from the directors of the two leading research institutions involved. The study was reviewed and approved by the Ethics Committee of the Medical Faculty at Ludwig Maximilian University of Munich (Project No. 23–0150), and no additional requirements were imposed regarding study procedures, informed consent, or data handling. Data access for research purposes was granted on the dates of March 2, 2024, and August 28, 2024- at no point during or after data collection did the authors have access to personally identifiable information (PII).

## Results

### Infectious disease prevalence in the full cohort and migration-history subgroup

**Fig 1:**
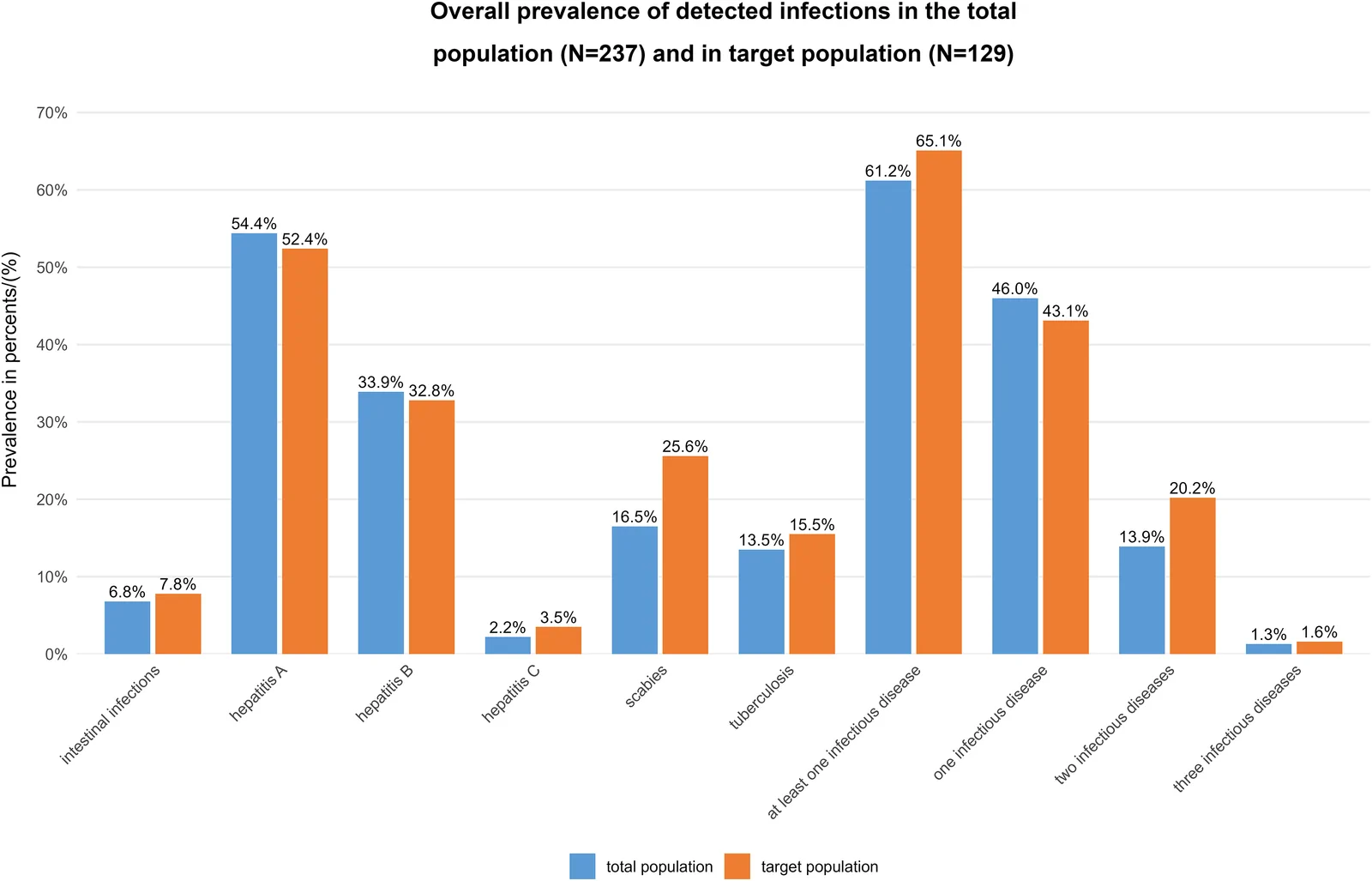
Overall prevalence of detected infections in the full cohort and migration-history subgroup.

The full study population comprised 237 unaccompanied minors examined by the Public Health Service between 2010 and 2022. Of these, 129 minors had sufficiently documented migration journey information and therefore formed the migration-history subgroup used for analyses involving journey characteristics. Fig 1 compares the prevalence of detected infections in the full cohort and the migration-history subgroup. Overall, the prevalence patterns were broadly similar between both populations. At least one infectious disease was detected in 61.2% of the full cohort and 65.1% of the migration-history subgroup. Hepatitis A was observed in 54.4% and 52.4%, hepatitis B in 33.9% and 32.8%, scabies in 16.5% and 25.6%, and tuberculosis in 13.5% and 15.5%, respectively. Two infectious diseases were detected in 13.9% of the full cohort and 20.2% of the subgroup, while three infectious diseases were uncommon in both populations. Of the 129 unaccompanied minors with documented migration journey information (54.4% of the full cohort of 237) 103 (79.8%) were male and between 13.5 and 16.5 years of age (55.0%). Nearly half (46.5%) were from Sub-Saharan Africa. Over half (52.9%, n = 65) transited through three or four countries. Most (72.1%) used two different modes of travel (air, land, and sea). The duration of the migration journey was under eight months for 65.1% of participants (Table 1).

**Table 1:** Socio-epidemiology of 129 unaccompanied minors.

Characteristic	Category	n (%)
**Age group (years)**	2.1-13.5	28 (21.7)
	13.5-16.5	71 (55.0)
	16.5-18.2	30 (23.3)
**Sex**	Girls	26 (20.2)
	Boys	103 (79.8)
**Region of origin**	Sub-Saharan Africa	60 (46.5)
	South Asia	36 (27.9)
	Middle East	19 (14.7)
	North Africa	6 (4.7)
	Southeast Europe	8 (6.2)
**Number of transit countries**	1	23 (18.7)
	2	30 (24.4)
	3	30 (24.4)
	4	35 (28.5)
	5	5 (4.1)
**Number of migration modes**	1 mode	35 (27.1)
	2 modes	93 (72.1)
	3 modes	1 (0.8)
**Migration journey duration**	<4 months	52 (41.3)
	4-8 months	30 (23.8)
	>8 months	44 (34.9)

Percentages may not total 100% in all categories because of missing or incomplete observations for selected variables.

The full cohort comprised all 237 unaccompanied minors examined by the Public Health Service between 2010 and 2022. The migration-history subgroup comprised 129 minors with sufficiently documented migration journey information. This subgroup was used for analyses involving migration journey characteristics.

### Infectious diseases

Infectious disease positivity was highest in the 13.5-16.5-year age group, where 74.7% (95% CI 62.7–83.9) tested positive for at least one screened infection. In contrast, prevalence was 64.3% (95% CI 44.1–80.7) among younger children (2.1–13.5 years) and 43.3% (95% CI 26.0–62.3) among older adolescents (16.5–18.2 years). Mean age did not differ substantially between infected (15.0 years, 95% CI 14.1–15.8) and uninfected participants (15.8 years, 95% CI 10.4–21.2).

Infection prevalence was 69.2% (95% CI 48.1–84.9) among girls and 64.1% (95% CI 54.0–73.1) among boys. Boys more commonly had single infections (46.6% vs 30.8%), whereas girls more often had two (30.8% vs 17.5%) or three (7.7% vs 0%) infections. Infection prevalence was high across all regions (57.9%−75.0%), though notable regional differences emerged. Intestinal parasitosis was less common in minors from South Asia (2.8%, 95% CI 0.2–16.2), compared to those from Sub-Saharan Africa, the Middle East, and Southeast Europe. Hepatitis A was least common among those from Sub-Saharan Africa (21.7%, 95% CI 12.5–34.5). Hepatitis B prevalence was markedly higher among North African (83.3%, 95% CI 36.5–99.1) and Southeast European participants (75.0%, 95% CI 35.6–95.6) – more than double rates observed in other regions. Hepatitis C, however, was only detected in minors from Southeast Europe (25.0%, 95% CI 4.5–64.4) and North Africa (16.7%, 95% CI 0.9–63.5).

Scabies was absent in unaccompanied minors from North Africa and the Middle East but was common in those from Sub-Saharan Africa (41.7%, 95% CI 29.3–55.1) and Southeast Europe (37.5%, 95% CI 10.2–74.1). Tuberculosis was a relevant concern among minors from Sub-Saharan Africa, with a prevalence of 20.0% (95% CI 11.2–32.7). In the migration-history subgroup, infectious disease positivity was numerically higher among minors with longer documented migration journeys, ranging from 51.0% (95% CI 36.5–65.4) for journeys shorter than four months to 76.6% (95% CI 61.6–87.2) for journeys longer than eight months. However, confidence intervals were wide and did not provide unambiguous statistical evidence of differences between journey-duration groups. Similar descriptive patterns were observed for hepatitis B and scabies, whereas hepatitis C and tuberculosis did not show consistent patterns across duration categories (Table 2).

**Table 2:** Prevalence of selected infectious diseases by socio-epidemiological and migration-related characteristics among 129 unaccompanied minors, % (95% CI).

Characteristic		Intestinal parasitosis	hepatitis A	hepatitis B	hepatitis C	Scabies	(Latent) tuberculosis	Infectious disease positive
**Age group (years)**	2.1-13.5	3.6 (0.2-20.2)	28.6 (14.0-48.9)	42.9 (25.0-62.6)	3.6 (0.2-20.2)	32.1 (16.6-52.4)	14.3 (4.7-33.6)	64.3 (44.1-80.7)
	13.5-16.5	8.5 (3.5-18.1)	32.4 (22.0-44.7)	32.4 (22.0-44.7)	2.8 (0.5-10.7)	26.8 (17.3-38.8)	15.5 (8.4-26.5)	74.7 (62.7-83.9)
	16.5-18.2	10.0 (2.6-27.7)	6.7 (1.2-23.5)	23.3 (10.6-42.7)	NA	16.7 (6.3-35.5)	10.0 (2.6-27.7)	43.3 (26.0-62.3)
**Sex**	Girls	3.9 (0.2-21.6)	23.1 (9.8-44.1)	46.2 (27.1-66.3)	7.7 (1.3-26.6)	30.8 (15.1-52.0)	19.2 (7.3-40.0)	69.2 (48.1-84.9)
	Boys	8.7 (4.3-16.4)	26.2 (18.3-36.0)	40.8 (31.3-50.9)	1.0 (0.1-6.1)	24.3 (16.6-33.9)	12.6 (7.2-21.0)	64.1 (54.0-73.1)
**Region of origin**	Sub-Saharan Africa	10.0 (4.1-21.2)	21.7 (12.5-34.5)	38.3 (26.4-51.8)	NA	41.7 (29.3-55.1)	20.0 (11.2-32.7)	68.3 (54.9-79.4)
	South Asia	2.8 (0.2-16.2)	30.6 (16.9-48.3)	38.9 (23.6-56.5)	NA	13.9 (5.2-30.3)	13.9 (5.2-30.3)	61.1 (43.5-76.4)
	Middle East	10.5 (1.8-34.5)	36.8 (17.2-61.4)	31.6 (13.6-56.5)	NA	NA	5.3 (0.3-28.1)	57.9 (34.0-78.9)
	North Africa	NA	33.3 (6.0-75.9)	83.3 (36.5-99.1)	16.7 (0.9-63.5)	NA	NA	66.7 (24.1-94.0)
	Southeast Europe	12.5 (0.7-53.3)	NA	75.0 (35.6-95.6)	25.0 (4.5-64.4)	37.5 (10.2-74.1)	NA	75.0 (35.6-95.6)
**Number of transit countries**	1	NA	13.8 (4.5-32.6)	31.0 (16.0-51.0)	3.5 (0.2-19.6)	10.3 (2.7-28.5)	3.5 (0.2-19.6)	38.0 (21.3-57.6)
	2	11.1 (3.6-27.0)	16.7 (7.0-33.5)	47.2 (30.8-64.3)	2.8 (0.2-16.2)	19.4 (8.8-36.6)	8.3 (2.2-23.6)	58.3 (40.9-74.0)
	3	8.3 (2.2-23.6)	11.1 (3.6-27.0)	27.8 (14.8-45.4)	2.8 (0.2-16.2)	13.9 (5.2-30.3)	8.3 (2.2-23.6)	47.2 (30.7-64.3)
	4	4.9 (0.9-17.8)	34.2 (20.6-50.7)	NA	NA	36.6 (22.6-53.1)	26.8 (14.8-43.2)	68.3 (51.8-81.4)
	5	9.1 (0.5-42.9)	9.1 (0.5-42.9)	NA	NA	27.3 (7.3-60.7)	0.0*	27.3 (7.3-60.7)
**Migration journey duration**	<4 months	4.1 (0.7-15.1)	22.5 (12.3-37.0)	36.7 (23.8-51.8)	NA	14.3 (6.4-27.9)	4.1 (0.7-15.1)	51.0 (36.5-65.4)
	4-8 months	7.7 (2.0-22.0)	18.0 (8.1-34.1)	33.3 (19.6-50.3)	5.1 (0.9-18.6)	18.0 (8.1-34.1)	10.3 (3.3-25.2)	53.9 (37.4-69.6)
	>8 months	10.6 (4.0-23.9)	27.7 (16.1-42.9)	48.9 (34.3-63.7)	2.1 (0.1-12.7)	40.4 (26.7-55.7)	4.1 (0.7-15.1)	76.6 (61.6-87.2)

NA indicates that the estimate was not available because the infection was not assessed in that subgroup or because the underlying data were insufficient or missing. Values of 0 indicate that no positive case was observed in the subgroup. *Confidence interval could not be calculated due to insufficient values. “Infectious disease positive” was defined as testing positive for at least one of the screened infections.

## Discussion

In this retrospective cross-sectional study, detected infections were common among unaccompanied minors examined by the PHS in one Bavarian district. The analysis showed broadly similar prevalence patterns between the full cohort and the migration-history subgroup. However, findings from the subgroup with migration journey information should be interpreted cautiously because several categories were small and formal comparisons did not provide unambigous statistical evidence of differences by journey duration. Infection prevalence peaked in the 13.5-16.5-year age group, with lower rates among younger children and older adolescents. Although overall infection prevalence was similar between sexes, girls more commonly had multiple infections – a finding requiring cautious interpretation given the small female sample size.

Regional disease patterns may help contextualize screening findings and clinical follow-up needs: 1. Intestinal parasitosis was found to be relatively uncommon among individuals from South Asia, suggesting lower observed prevalence in this subgroup. 2. Hepatitis A, B, and C were identified across all regions, underscoring the need for universal screening and prevention strategies regardless of region of origin. 3. Scabies was not detected in refugees from North Africa and the Middle East, suggesting it may be a less pressing issue in these populations. 4. Tuberculosis affected one in five Sub-Saharan African participants (20.0%, 95% CI 11.2–32.7), suggesting this subgroup may benefit from targeted screening in similar settings. However, generalizability to other regions or time

periods is uncertain. Infectious disease positivity was numerically higher among minors with longer documented migration journeys, but this pattern was not statistically unambigous and may reflect confounding by factors such as region of origin, living conditions during transit, access to healthcare, or differences in screening indications.

### Burden of infections

Serological testing for hepatitis A has only been conducted in a limited number of studies involving unaccompanied minors. Kloning et al., for example, reported hepatitis A immunity in 92.8% of a group of 154 unaccompanied minors in two German outpatient care facilities [28]. In contrast, our study found a lower prevalence of up to 40.0% (95% CI 24.4–57.8). This discrepancy is likely due to differences in the study populations and testing strategies: while Kloning et al. tested all participants systematically [28], our hepatitis A analyses were performed only when medically indicated, potentially introducing selection bias. Hepatitis B prevalence among North African participants (83.3%, 95% CI 36.5–99.1) substantially exceeded the 7.9% reported by Spallek et al. [7]. Our findings encompass the 62.4% prevalence reported by Hampel et al. [15]. In comparison, much lower prevalence rates were observed by Janda et al. [29] (7.7%), Williams et al. [30] (4.8%), and Cardoso Pinto et al. [31] (4.6%). Notably, Kloning et al. reported hepatitis B immunity in 31.8% of cases [28], which closely matches our estimate of 31.6% (95% CI 13.6–56.5) among unaccompanied minors from the Middle East. Hepatitis C prevalence estimates were higher than expected in some very small subgroups, but these findings should be interpreted cautiously because of limited precision and lack of comparable data. A prevalence of up to 50.0% (95% CI 9.5–90.6) among unaccompanied minors who used three modes of migration contrasts starkly with the 0.5% reported by Williams et al. [30]. The lack of comparable data in other studies makes this disparity difficult to reconcile and warrants further investigation. Scabies prevalence in the oldest age group (16.7%, 95% CI 6.3–35.5) was consistent with prior reports from Frankfurt (16%) [32] and Janda et al. (14.2%) [29].

Regarding tuberculosis, our age-group-specific prevalence of 10.0% to 15.5% aligns with findings from the Augsburg Public Health Department (13.0%) [22], Berlin (13.9%) [33], Frankfurt am Main (21%) [32], and Stuttgart (17.3%) [22]. The prevalence we observed for unaccompanied minors from Sub-Saharan Africa, 20.0% (95% CI 11.2–32.7), and for those with four transit countries, 26.8% (95% CI 14.8–43.2), is consistent with the 23% reported by Williams et al. [30]. The markedly lower prevalence reported by Cardoso Pinto et al. [31] (1.3%) is comparable to the 3.5% (95% CI 0.2–19.6) in our cohort with only one transit country, possibly reflecting differences in exposure or screening intensity.

Nearly all of our intestinal parasitosis prevalence estimates were lower than the 19.6% reported in Bielefeld [34]. However, the 3.6% (95% CI 0.2–20.2) observed in children aged 2.1 to 13.5 years was consistent with the 3.5% reported across the state of Bavaria [14]. The literature again shows a heterogeneous picture: 16.0% in Williams et al. [30], 17.5% in Frankfurt [32], and 6.7% in Janda et al. [29], likely due to regional and demographic differences in study cohorts. HIV prevalence was extremely low, consistent with state-wide trends in Bavaria, where reactive HIV tests among asylum seekers over 15 years remained below 1% between 2013 and 2015 (0.3% in 2015, 0.6% in 2016) [13,14]. Mohammadzadeh et al. also reported only six HIV infections among 16,601 refugees in Bremen between 2011 and 2014 [35]. The complete absence of HIV infections in our study cohort is therefore consistent with these data. Similarly, Kloning et al. [28] and Williams et al. [30] found no HIV cases, while Cardoso Pinto et al. [31] reported a marginal rate of 1.3%. Finally, the proportion of unaccompanied minors with at least one infectious disease in the 2.1 to 13.5-year age group, 53.5% (95% CI 41.4–65.3), corresponds closely with the 58.8% reported by Spallek et al. [7] and the 48.6% by Laukamp et al. [34], reinforcing the consistency of findings across studies.

### Migration journey specific prevalences of infectious diseases

To our knowledge, few studies have examined infection prevalence in relation to detailed migration journey characteristics among unaccompanied minors. In our migration-history subgroup, several regional and journey-related prevalence patterns were observed. Hepatitis B and C prevalence was highest among minors from North Africa and Southeast Europe, whereas scabies and tuberculosis were more frequently observed among minors from Sub-Saharan Africa. The number of transit countries did not show a consistent pattern across specific infections. Overall infectious disease positivity was numerically higher among minors who transited through up to four countries, but prevalence was lower in the small subgroup with five transit countries. Similarly, infectious disease positivity was numerically higher among minors with longer migration journeys. However, these descriptive differences were not statistically unambiguous, confidence intervals were wide, and several subgroup estimates were based on small numbers. The findings should therefore be interpreted as hypothesis-generating rather than evidence of a confirmed association between migration journey characteristics and infectious disease positivity.

### Strengths and weaknesses of the study

A strength of this study is the availability of standardized health assessment data from all unaccompanied minors examined in the region over a 13-year period, with detailed migration-history information available for a substantial subgroup. The continuity of the study team throughout the observation period further supports consistency in data collection and interpretation. In addition, comparing infection prevalence in the full cohort and the migration-history subgroup allowed us to assess the relationship between the two populations. Several limitations should be acknowledged. Although overall prevalence patterns were broadly similar, the subgroup had somewhat higher prevalence of scabies, tuberculosis, and multiple infections. Therefore, migration-specific findings may not be fully representative of all 237 minors examined during the study period. Self-reported migration journey data may be subject to recall bias, and language barriers may have led to misclassification or underreporting despite translation support. The long study period may obscure shorter-term changes in migration routes, healthcare access, or conflict dynamics. The restriction to minors with documented migration history (54.4% of the full cohort) may have introduced selection bias. In addition, age-dependent screening protocols limit direct comparisons across age groups. Finally, the limited sample size, several very small subgroups, and the single-district setting restrict statistical precision and generalizability beyond this regional and temporal context.

## Conclusions

Detected infections were common among unaccompanied minors examined in this Bavarian district, both in the full cohort and in the subgroup with documented migration histories. In the migration-history subgroup, infectious disease positivity was numerically higher among minors with longer documented migration journeys, but this pattern was not statistically unambigous and should be interpreted cautiously given wide confidence intervals, small subgroup sizes, and potential confounding. Structured health screening after arrival remains important, and migration history may provide useful contextual information during clinical assessment.
